# Innate immunity but not NLRP3 inflammasome activation correlates with severity of stable COPD

**DOI:** 10.1136/thoraxjnl-2012-203062

**Published:** 2014-01-15

**Authors:** Antonino Di Stefano, Gaetano Caramori, Adam Barczyk, Chiara Vicari, Paola Brun, Andrea Zanini, Francesco Cappello, Elvira Garofano, Anna Padovani, Marco Contoli, Paolo Casolari, Andrew L Durham, Kian Fan Chung, Peter J Barnes, Alberto Papi, Ian Adcock, Bruno Balbi

**Affiliations:** 1 Divisione di Pneumologia e Laboratorio di Citoimmunopatologia dell'Apparato Cardio Respiratorio, Fondazione Salvatore Maugeri, IRCCS, Veruno (NO) e Tradate, Pavia, Italy; 2 Centro Interdipartimentale per lo Studio delle Malattie Infiammatorie delle Vie Aeree e Patologie Fumo-correlate (CEMICEF; formerly Centro di Ricerca su Asma e BPCO), Sezione di Medicina Interna e Cardiorespiratoria (formerly Sezione di Malattie dell'Apparato Respiratorio), Università di Ferrara, Ferrara, Italy; 3 Katedra i Klinika Pneumonologii Slaskiego Uniwersytetu Medycznego w Katowicach, Slaskiego, Poland; 4 Department of Molecular Medicine, Histology Unit, University of Padova, Padova, Italy; 5 Dipartimento di Biomedicina Sperimentale e Neuroscienze Cliniche, Sezione di Anatomia Umana, Università di Palermo, Palermo, Italy; 6 Istituto Euro-Mediterraneo di Scienza e Tecnologia, Palermo, Italy; 7 Istituto Paolo Sotgiu, Libera Università degli Studi di Scienze Umane e Tecnologiche, Lugano, Switzerland; 8 Airway Disease Section, National Heart and Lung Institute, Imperial College London, London, UK

**Keywords:** COPD Pathology, Innate Immunity

## Abstract

**Background:**

In models of COPD, environmental stressors induce innate immune responses, inflammasome activation and inflammation. However, the interaction between these responses and their role in driving pulmonary inflammation in stable COPD is unknown.

**Objectives:**

To investigate the activation of innate immunity and inflammasome pathways in the bronchial mucosa and bronchoalveolar lavage (BAL) of patients with stable COPD of different severity and control healthy smokers and non-smokers.

**Methods:**

Innate immune mediators (interleukin (IL)-6, IL-7, IL-10, IL-27, IL-37, thymic stromal lymphopoietin (TSLP), interferon γ and their receptors, STAT1 and pSTAT1) and inflammasome components (NLRP3, NALP7, caspase 1, IL-1β and its receptors, IL-18, IL-33, ST2) were measured in the bronchial mucosa using immunohistochemistry. IL-6, soluble IL-6R, sgp130, IL-7, IL-27, HMGB1, IL-33, IL-37 and soluble ST2 were measured in BAL using ELISA.

**Results:**

In bronchial biopsies IL-27+ and pSTAT1+ cells are increased in patients with severe COPD compared with control healthy smokers. IL-7+ cells are increased in patients with COPD and control smokers compared with control non-smokers. In severe stable COPD IL-7R+, IL-27R+ and TSLPR+ cells are increased in comparison with both control groups. The NALP3 inflammasome is not activated in patients with stable COPD compared with control subjects. The inflammasome inhibitory molecules NALP7 and IL-37 are increased in patients with COPD compared with control smokers. IL-6 levels are increased in BAL from patients with stable COPD compared with control smokers with normal lung function whereas IL-1β and IL-18 were similar across all groups.

**Conclusions:**

Increased expression of IL-27, IL-37 and NALP7 in the bronchial mucosa may be involved in progression of stable COPD.

**Key messages**

**What is the key question?**

- Are innate immunity and/or inflammasome activation in the upper and lower airways involved in the progression of severity of stable COPD?

**What is the bottom line?**

- In our study the increased expression of the innate immunity inflammatory mediators interleukin (IL)-6, IL-27, IL-37 and NALP7 in the bronchial mucosa and/or bronchoalveolar lavage may be involved in the progression of the severity of stable COPD.

**Why read on?**

- The lack of evidence for inflammasome activation in the upper and lower airways of patients with stable COPD suggests that this inflammatory pathway is not involved in the progression of the severity of stable COPD.

## Introduction

Inflammation is important in the pathogenesis of stable COPD.^1^ Environmental stress such as cigarette smoke activates the innate immune response which may drive COPD inflammation.^2^ Interleukin 6 (IL-6) is a multifunctional pro-inflammatory cytokine^1^ that acts via two molecules: the IL-6R (IL-6 receptor) and gp130.^3^ Soluble gp130 (sgp130) inhibits IL-6 trans-signalling via the soluble IL-6R (sIL-6R) and classic signalling via the membrane bound IL-6R (mIL-6R).^4^

IL-27, an IL-12/IL-23 family member, stimulates T helper 1 (Th1) lymphocyte differentiation.^5^ It also stimulates haematopoiesis, increases antigen presentation by antigen-presenting cells and inhibits angiogenesis.^6^ The IL-27 receptor (IL-27Ra or WSX-1) activates the Janus kinase (JAK) pathway with phosphorylation of signal transducer and activator of transcription (STAT)-1 and STAT3.^6^ IL-10, in contrast, potently inhibits the expression of inflammatory proteins such as IL-1β, tumour necrosis factor α and matrix metalloproteinase 9.^1^

IL-7 is primarily produced by stromal and epithelial cells^7^ ^8^ and promotes human T-cell development, naïve T-cell homeostasis, T-cell proliferation and survival of memory T cells.^7^ ^8^ IL-7 binds to the IL-7R, a heterodimer consisting of the IL-7Rα (CD127) and the common γ chain (γc or CD132), causing STAT1 and STAT3 activation.^7^ Thymic stromal lymphopoietin (TSLP) is an IL-7 family member involved in the activation, expansion and survival of T lymphocytes and dendritic cells acting through a heterodimeric IL-7Rα and TSLPR complex.^9^

High mobility group box 1 (HMGB1) is a nuclear protein that can act as a damage-associated molecular pattern to activate immune cells, including Th1 lymphocytes.^10^

NLRs are categorised into five subfamilies.^11^ ^12^ A typical inflammasome is composed of an NLR, an adaptor protein such as apoptosis-associated speck-like protein containing a CARD (ASC) and an effector caspase that activates proinflammatory cytokines. Within the NRLP3 complex, auto-catalytic cleavage of pro-caspase 1 enables removal of IL-1β and IL-18 pro sequences resulting in biologically active forms and thereby initiating Th1 and Th17 adaptive immune responses.^11^ ^13^ HMGB1 is also induced by NRLP3 activation.^11^ ^12^ The expression of IL-33, another member of the IL-1 family, may also be enhanced through activation of the inflammasome.^14^ IL-33 acts through its receptor ST2 and may activate immune cells.^15^ IL-6 is induced by NRLP3 but in an inflammasome-independent manner.^16^

The NLRP3 inflammasome is activated by infectious agents, double-strand DNA and extracellular ATP.^2^ ^11^ NALP7 decreases transcription of pro-IL-1β expression and IL-10 and IL-27 can modulate NRLP3 activity^2^ whereas IL-37 can downregulate inflammation and innate immunity independently of the inflammasome.^17^ ^18^

The aim of this study was to investigate the presence of innate immune mediators (IL-6, IL-7, IL-10, IL-27, IL-37, TSLP, interferon γ (IFNγ), their receptors, and signalling proteins STAT1 and pSTAT1) and inflammasome components (NLRP3, caspase 1, IL-1β, IL-18, IL-33, NALP7, ST2) in the bronchial mucosa and bronchoalveolar lavage (BAL) of patients with stable COPD of differing severity and age-matched control subjects with normal lung function.

## Methods

### Subjects

All subjects were recruited from the Respiratory Medicine Unit of the ‘Fondazione Salvatore Maugeri’ (Veruno, Italy), the Section of Respiratory Diseases of the University Hospital of Ferrara, Italy and the Section of Respiratory Diseases of the University Hospital of Katowice, Poland for immunohistochemistry and ELISA experiments. The severity of the airflow limitation, as determined by spirometry, was graded using Global Initiative for Chronic Obstructive Lung Disease (GOLD) criteria.^19^ All former smokers had stopped smoking for at least 1 year. COPD and chronic bronchitis were defined, according to international guidelines: COPD, presence of a post-bronchodilator forced expiratory volume in 1s (FEV_1_)/forced vital capacity ratio <70%; chronic bronchitis, presence of cough and sputum production for at least 3 months in each of two consecutive years (http://www.goldcopd.com). All patients with COPD were stable. The study conformed to the Declaration of Helsinki. We obtained and studied bronchial biopsies from 55 subjects: 32 had a diagnosis of COPD in a stable clinical state,^20^ 12 were current or ex-smokers with normal lung function, and 11 were non-smokers with normal lung function (table 1). The smoking history was similar in the three smoker groups: mild/moderate and severe/very severe COPD, and healthy smokers with normal lung function. Clinical details of the patients in whom BAL was collected are summarised in table 2. The results provided are the data from 26 patients with COPD and 18 control smokers with normal lung function. Due to the necessity to concentrate the BAL supernatants the results provided for each ELISA are the data from 15 patients with COPD and 14 control smokers with normal lung function which are not the same patients for all mediators measured.

**Table 1:** Clinical characteristics of subjects studied by immunohistochemistry

	Control non-smokers	Control smokers normal lung function	COPD grade I/II (mild/moderate)	COPD grade III/IV (severe/very severe)
Number	11	12	14	18
Age (years)	67±1	61±7	67±8	66±9
M/F	10/1	9/3	12/2	11/7
Pack years	0	43±26	40±19	54±36
Ex/current smokers	0	2/10	5/9	13/5
FEV_1_ pre-β_2_ (% predicted)	116±14	104±13	66±14*	35±8*^,^**
FEV_1_ post-β_2_ (% predicted)	ND	ND	72±12	38±9
FEV_1_/FVC (%)	85±10	81±6	60±8*	44±10*^,^**
Chronic bronchitis	0	5	8	6

Patients were classified according to Global Initiative for Chronic Obstructive Lung Disease (GOLD) (http://www-goldcopd.org) grades of severity for COPD into mild (stage I), moderate (stage II), severe (stage III) and very severe (stage IV).

Patients with COPD were using short-acting inhaled β_2_ agonists (SABAs) or short-acting inhaled antimuscarinics (SAMAs) as needed or regular long-acting inhaled β_2_ agonists (LABAs) and/or regular inhaled anticholinergics, including SAMA or long-acting inhaled antimuscarinics at the dosage recommended in current COPD guidelines (http://www.goldcopd.org) at the time of their recruitment.

Data expressed as means±SEM. For patients with COPD FEV_1_/FVC (%) are post-bronchodilator values.

Statistics (ANOVA): *p<0.0001, significantly different from control smokers with normal lung function and control never smokers; **p<0.0001, significantly different from patients with mild/moderate COPD.

F, female, FEV_1_, forced expiratory volume in 1s; FVC, forced vital capacity; M, male; ND, not determined.

**Table 2:** Clinical characteristics of the subjects for the BAL study

	Smokers with normal lung function	Patients with mild to moderate stable COPD
Number	18	26
Age	64.4±2.0	67.9±1.6
Sex (M/F)	15/3	19/F
Ex/current smokers	10/8	12/14
Pack-years	37.8±3.0	39.6±5.5
Chronic bronchitis	10	14
FEV_1_ % predicted	88.2±4.9	57.9±3.0
FEV_1_/FVC %	79.2±1.8	56.8±2.5

FEV_1_ % predicted and FEV_1_/FVC % are post-bronchodilator values. Patients with COPD were using short-acting inhaled β_2_ agonists or short-acting inhaled antimuscarinics (SAMAs) as required or regular long-acting inhaled β_2_ agonists and/or regular inhaled anticholinergics, including SAMAs or long-acting inhaled antimuscarinics at the dosage recommended in current COPD guidelines (http://www.goldcopd.org) at the time of their recruitment. Data expressed as means±SEM.

BAL, bronchoalveolar lavage; F, female; FEV_1_, forced expiratory volume in 1s; FVC, forced vital capacity; M, male.

A detailed description of subjects, lung function tests, fibreoptic bronchoscopy and processing of bronchial biopsies and BAL, immunohistochemistry, scoring system for immunohistochemistry, double staining and confocal microscopy, ELISA tests performed on the BAL fluid and ‘in vitro’ experiments performed on normal human bronchial epithelial (NHBE) cells and details of statistical analysis are provided in the online supplementary data repository.

### Statistical analysis

Differences between groups were analysed using analysis of variance and Kruskal–Wallis tests. Correlation coefficients were calculated using the Spearman rank method.

## Results

### Measurement of inflammatory cells in the bronchial submucosa

These results are reported in full in the online supplementary data repository and in table E1. Briefly, these data confirm elevated numbers of CD8 T cells, CD68 macrophages and neutrophils in patients with COPD.^20^

### Immunohistochemistry for innate immunity and inflammasome pathways in the bronchial epithelium

**Figure 1:**
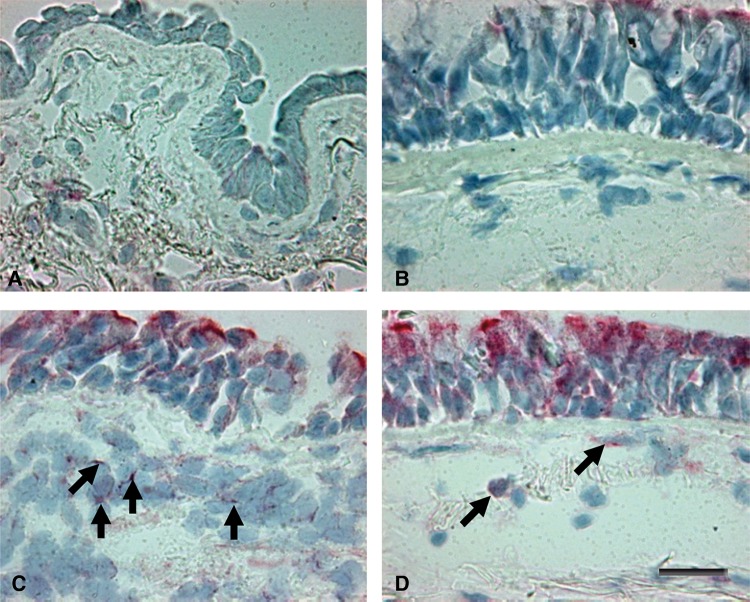
Photomicrographs showing the bronchial mucosa from (A) control non-smoker, (B) control healthy smoker with normal lung function, (C) patient with mild/moderate stable COPD and (D) patient with severe stable COPD immunostained for identification of IL-27+ cells (arrows) in the epithelium and bronchial submucosa. Results are representative of those from 11 non-smokers, 12 healthy smokers, 14 patients with mild/moderate COPD and 18 patients with severe COPD. Bar=30 μm.

The number of IL-27+ (figure 1 and table 3), IL-27R+, TSLPR+, NALP7 and STAT1 immunoreactive cells was increased in the epithelium of patients with severe stable COPD compared with control non-smokers (p=0.0048, p=0.017, p=0.008, p=0.054, p=0.011, respectively). IL-27+, TSLPR+, NALP7+ and STAT1+ cells also differed significantly in comparison with control healthy smokers (p=0.043, p=0.0019, p=0.0009 and p=0.023, respectively) (table 3). In contrast, no significant differences in bronchial epithelial expression of IL-1β, IL-1βRI and RII, caspase-1, IL-18, IL-18Rα, IL-18Rβ, IL-18BP, NLRP3, IL-6, IL-6Rα, IL-7, IL-7Rα, IL-10, IL-10Rα, IL-33, ST-2, IFNγ, IFNγRI, pSTAT1, TSLP and IL-37 were observed between groups (table 3). Due to the fewer number of subjects who were current smokers within the severe stable COPD group we were unable to perform subanalysis of current versus former smokers across groups.

**Table 3:** Immunohistochemical quantification of innate immunity and inflammasome pathways expression in the bronchial mucosa

	Healthy non-smokers	Healthy smokers	Patients with mild/moderate COPD	Patients with severe COPD	p Value
Epithelium (score 0–3)					
IL-6	0.25 (0–0.5)	0.25 (0–0.5)	0.25 (0–1)	0.25 (0–1)	0.346
IL-6 Rα	0 (0–0)	0 (0–0)	0 (0–0)	0 (0–0)	n.v.
IL-7	0.5 (0.5–1)	0.75 (0.25–2)	0.5 (0.25–1.5)	0.75 (0.5–2)	0.671
IL-7 Rα	0 (0–0)	0 (0–0)	0 (0–0.5)	0 (0–0.5)	0.985
IL-10	0 (0–0.25)	0 (0–0.25)	0 (0–0.5)	0 (0–0.5)	0.513
IL-10 Rα	0 (0–0.25)	0 (0–0.25)	0 (0–0.25)	0 (0–0.25)	0.688
IL-27	0.5 (0.25–1)	0.75 (0.25–1.5)	0.75 (0–1.5)	1 (0.5–2)*^,^**	0.0279
IL-27 R	0 (0–0.5)	0.25 (0–0.5)	0.25 (0–0.75)	0.25 (0–1)*	0.0748
IL-33	0 (0–0.25)	0 (0–0.25)	0 (0–0.25)	0 (0–0.75)	0.962
ST2	0 (0–0)	0 (0–0)	0 (0–0.25)	0 (0–0.25)	0.752
TSLP	0.25 (0–1)	0.25 (0–1)	0.5 (0–1)	0.37 (0–1)	0.545
TSLP-R	0.25 (0.25–0.5)	0.25 (0.25–0.5)	0.5 (0.25–1.5)	1 (0.25–1.25)*^,^**	0.007
IL-18	1 (0.5–2)	1 (0.5–2.5)	1.5 (0.25–2.5)	1 (0.5–2.5)	0.386
IL-18 Rα	2.5 (0.75–2.75)	2.5 (1–3)	2.5 (1–3)	2.5 (1–3)	0.869
IL-18 Rβ	2.25 (1–2.5)	1 (0.25–2.5)	1.5 (0–2.5)	2 (1–2.75)	0.095
IL-18 BP	0 (0–0)	0 (0–0.25)	0 (0–0.5)	0 (0.25)	0.093
NLRP-3	1.62 (1–2)	1.5 (1–2.75)	1.5 (1–3)	2 (1.5–2.5)***	0.220
Caspase-1	0 (0–0)	0 (0–0)	0 (0–0)	0 (0–0)	n.v.
IL-1β	0 (0–0.25)	0 (0–0.25)	0 (0–0.25)	0 (0–0.5)	0.671
IL-1β RI	0 (0–0)	0 (0–0)	0 (0–0)	0 (0–0)	n.v.
IL-1β RII	0 (0–0.25)	0.25 (0–0.5)	0.25 (0–1)	0.25 (0–1)	0.380
IL-37	1.5 (0.25–2.25)	1.5 (0.25–2.25)	1.12 (0.75–2.0)	1.5 (0.75–2.25)	0.915
NALP7	1.25 (0.5–2.5)	0.62 (0.25–1.75)	1.62 (0.25–2.5)	1.87 (0.5–3.0)*^,^**	0.006
STAT1	0.37 (0.25–1.5)	0.5 (0.0–2.0)	1.0 (0.25–2.25)	1.37 (0.25–2.75)*^,^**	0.018
pSTAT1	0 (0–0)	0 (0–0)	0 (0–0)	0 (0–0)	n.v.
IFN-γ	0 (0–0.25)	0 (0–0.25)	0 (0–0)	0 (0–0.25)	0.938
IFN-γ RI	1.5 (0.75–2.25)	1.25 (0.75–2.5)	1.25 (0.75–3)	2.25 (0.75–2.5)	0.282
Submucosa (cells/mm^2^) median (range)					
IL-6	24 (9–54)	27.5 (8–64)	36.5 (4–126)	44 (6–169)	0.294
IL-6 Rα	0 (0–22)	11 (0–55)	7 (0–18)	10 (0–37)	0.259
IL-7	72 (32–226)	126 (56–269)*	140 (32–484)*	140 (52–323)*	0.023
IL-7 Rα	13 (0–71)	32 (6–108)	44.5 (5–183)	91 (16–302)*^,^**^,^***	0.0053
IL-10	0 (0–12)	7 (0–28)*	6.5 (0–113)*	9 (0–55)*	0.0519
IL-10 Rα	0 (0–4)	0 (0–8)	0 (0–48)	0 (0–23)	0.367
IL-27	54 (8–113)	51 (14–140)	73 (9–400)	77 (36–542)**	0.113
IL-27 R	12 (0–24)	9 (0–51)	35 (0–81)*^,^**	48 (6–155)*^,^**	0.0027
IL-33	5 (0–9)	6 (0–23)	5 (0–54)	6 (0–86)	0.606
ST-2	0 (0–5)	0 (0–8)	0 (0–16)	0 (0–7)	0.722
TSLP	14 (0–29)	11 (0–38)	17 (0–90)	12 (0–81)	0.498
TSLP-R	18 (5–43)	12 (0–86)	55 (0–277)**	49 (7–269)*^,^**	0.011
IL-18	123 (11–203)	59 (16–355)	170 (16–419)	107 (8–488)	0.381
IL-18 Rα	177 (61–203)	152 (81–242)	231 (71–424)	214 (59–525)	0.118
IL-18 Rβ	138 (90–210)	69 (5–236)	104 (5–232)	182 (48–302)**^,^***	0.043
IL-18 BP	5 (0–97)	0 (0–32)	0 (0–18)	0 (0–16)	0.258
NLRP-3	104 (73–183)	111 (41–274)	166 (56–348)	121 (84–403)	0.261
Caspase-1	0 (0–0)	0 (0–0)	0 (0–0)	0 (0–0)	n.v.
IL-1β	0 (0–5)	6 (0–24)	5 (0–56)	4 (0–89)	0.298
IL-1β RI	0 (0–7)	0 (0–7)	0 (0–16)	0 (0–14)	0.362
IL-1β RII	0 (0–11)	0 (0–16)	0 (0–18)	0 (0–26)	0.882
IL-37	145 (43–258)	107 (58–355)	282 (113–441)*^,^**	213 (64–452)**	0.024
NALP7	167 (29–270)	85 (11–242)	210 (32–462)**	177 (45–456)**	0.005
STAT1	35 (19–102)	22 (13–129)	110 (9–210)*^,^**	129 (8–274)*^,^**	0.005
pSTAT1	37 (0–156)	8 (0–127)	41 (0–158)	67 (0–173)**	0.0887
IFN-γ	12 (0–46)	14.5 (0–115)	20 (3–90)*	22 (4–82)*	0.051
IFN-γRI	146 (118–298)	183 (113–460)	226 (129–672)	344 (170–631)*^,^**^,^***	0.0046

Data expressed as median (range). Statistics: the Kruskal–Wallis test was used for multiple comparisons followed by the Mann–Whitney U test for comparison between groups: *p<0.05, significantly different from control non-smokers; **p<0.05, significantly different from control smokers with normal lung function; ***p<0.05, significantly different from patients with mild COPD. The exact ‘p’ values for comparison between groups are given in the Results section.

IFN, interferon; IL, interleukin; n.v., no value; TSLP, thymic stromal lymphopoietin.

### Immunohistochemistry for innate immunity and inflammasome pathways in the bronchial submucosa

Mononuclear cells (lymphocytes and macrophages) and endothelial cells were the most represented immunostained cells in the submucosa. The number of IFNγ+ cells was significantly higher in patients with mild/moderate (p=0.010) and severe (p=0.008) stable COPD compared with control non-smokers, confirming previously reported data.^21^ The number of IFNγRI+ cells was increased in patients with severe COPD compared with patients with mild COPD (p=0.031), control smokers (p=0.0035) and control non-smokers (p=0.006). IL-18Rβ showed a slight increase in patients with severe COPD compared with those with mild/moderate COPD (p=0.045) and control smokers (p=0.039) but did not differ in comparison with control non-smokers. The number of IL-7+ (see online supplementary figure E1) cells was higher in patients with severe COPD (p=0.008), patients with mild/moderate COPD (p=0.010) and in control smokers (p=0.012) compared with control non-smokers. In addition, the number of IL-7Rα+ cells was significantly higher in patients with severe COPD compared with patients with mild/moderate COPD (p=0.040), control smokers (p=0.009) and control non-smokers (p=0.002). IL-10 was poorly expressed but the number of IL-10+ cells was higher in patients with severe stable COPD (p=0.005), patients with mild/moderate COPD (p=0.047) and in control smokers (p=0.054) compared with control non-smokers.

The number of IL-27+ (figure 1) and pSTAT1+ (see online supplementary figure E2) cells was significantly higher in patients with severe COPD (p=0.032 and p=0.018, respectively) compared with control smokers but did not differ in comparison with the other groups. Interestingly, the number of IL-27R+ cells was significantly higher in patients with severe COPD (p=0.010 and p=0.002) and patients with mild/moderate COPD (p=0.054 and p=0.009) compared with control smokers and non-smokers. Similarly, the number of total STAT1+ cells was significantly higher in patients with severe COPD (p=0.0043 and p=0.015) and patients with mild/moderate COPD (p=0.022 and p=0.029) compared with control smokers and non-smokers.

The number of TSLPR+ cells was higher in patients with severe COPD compared with control smokers (p=0.005) and non-smokers (p=0.044). TSLPR+ cell numbers were also increased in patients with mild/moderate stable COPD with control smokers (p=0.013).

**Figure 2:**
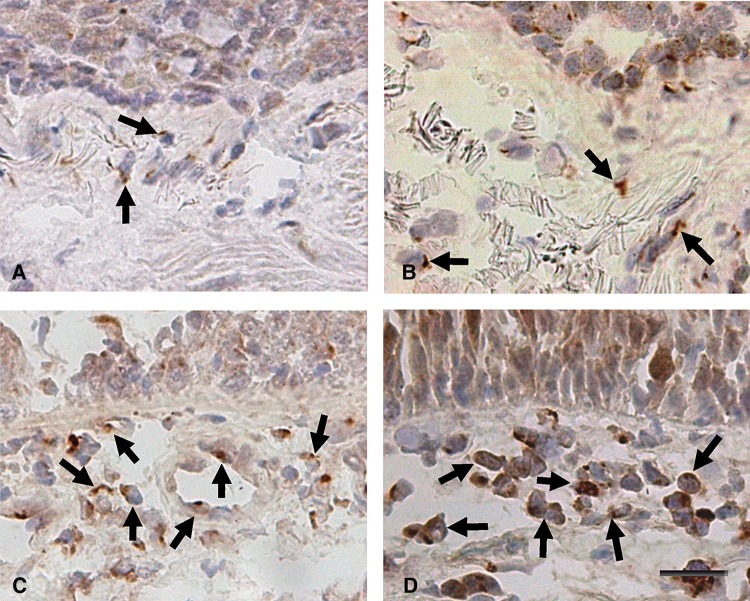
Photomicrographs showing the bronchial mucosa from (A) control non-smoker, (B) control healthy smoker with normal lung function, (C) patient with mild/moderate stable COPD and (D) patient with severe stable COPD immunostained for identification of (interleukin-37)+ cells (arrows) in the bronchial submucosa. Results are representative of those from 11 non-smokers, 12 healthy smokers, 14 patients with mild/moderate COPD and 18 patients with severe COPD. Bar=30 μm.

The number of IL-37+ (figure 2) and NALP7+ (see online supplementary figure E3) cells was higher in patients with severe stable COPD compared with control smokers (p=0.054 and p=0.0015 respectively) and in patients with mild/moderate stable COPD in comparison with control smokers (p=0.008 and p=0.0043 respectively). Furthermore, the number of IL-37+ cells in patients with mild/moderate stable COPD was significantly increased in comparison with control non-smokers (p=0.023) (table 3).

No significant differences were observed for IL-1β, IL-1βRI, IL-1βRII, caspase-1, IL-18, IL-18Rα, IL-18BP, NLRP3, IL-6, IL-6Rα, IL-10Rα, IL-33, ST-2 and TSLP immunostaining between groups.

### Double staining and confocal microscopy

The percentage of CD68+IL-27+ double-stained cells was significantly increased in patients with COPD (34±8%) compared with control smokers (8±2%, p=0.0209) (see online supplementary figure E4 and data repository for more details).

### ELISA assays in the BAL supernatants

**Figure 3:**
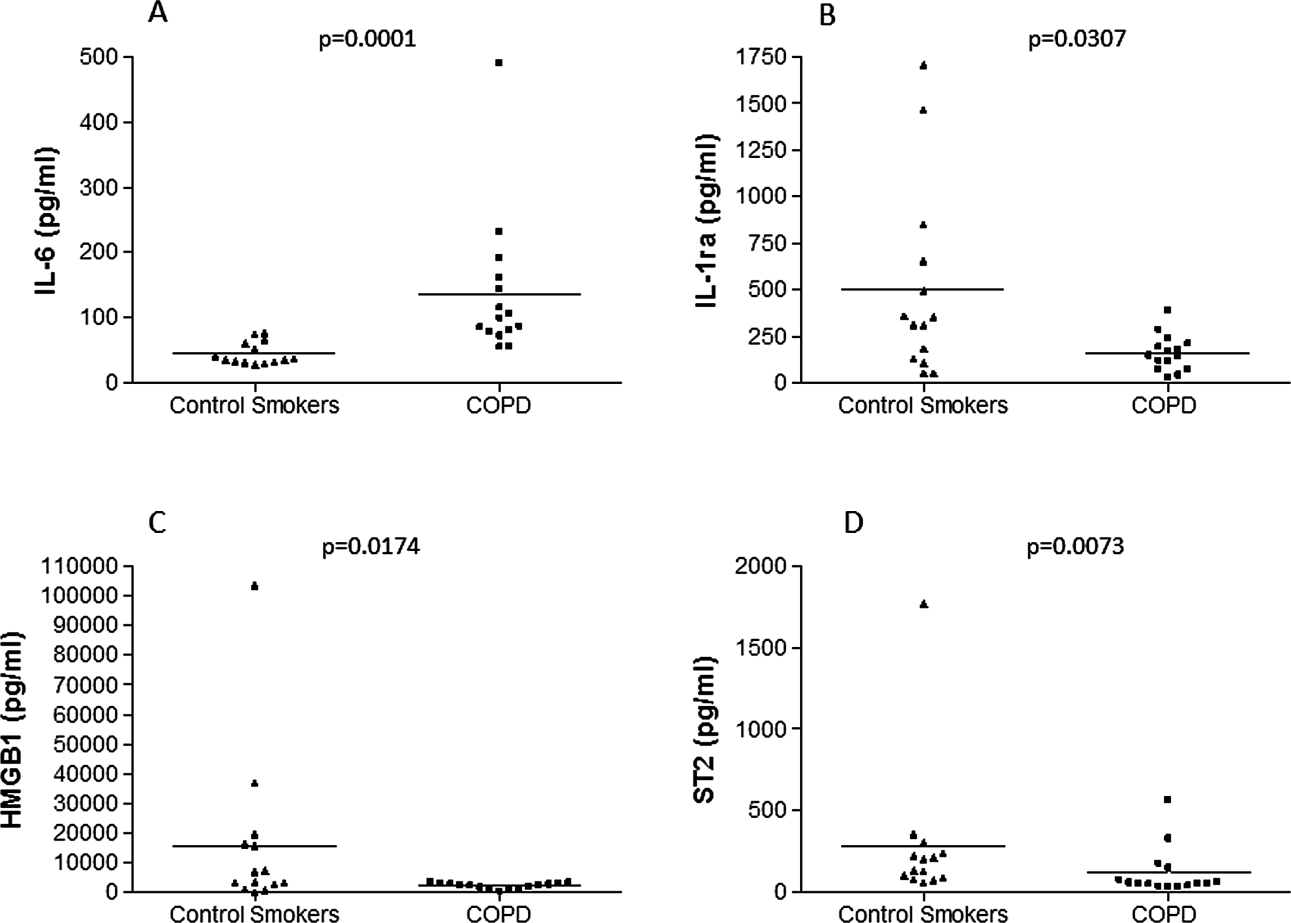
Bronchoalveolar lavage levels of (A) interleukin (IL)-6, (B) IL-1RA, (C) HMGB1 and (D) soluble ST2 in patients with stable COPD (n=15) compared with control healthy smokers (n=14). Results are expressed as mean±SEM. Exact p values are shown above each graph.

BAL levels of IL-6 were significantly increased in patients with stable COPD compared with the control healthy smokers (p=0.0001; figure 3A), without a significant change in the BAL level of sIL-6R and sgp130 between the two groups (see online supplementary figure E5A and E6A). In contrast, BAL IL-7 (see online supplementary figure E5B) and IL-27 (see online supplementary figure E5C) levels were not significantly different between the two subject groups. BAL HMGB1 was significantly decreased in patients with stable COPD compared with control healthy smokers (p=0.0174; figure 3C). However, this difference was lost after removal of the outliers (p=0.0540). The BAL level of soluble ST2 (p=0.0073; figure 3D) and IL-1RA (p=0.0307; figure 3B) are significantly decreased in patients with stable COPD compared with control smokers with normal lung function without significant changes in the BAL level of IL-1β (see online supplementary figure E6B), IL-18 (see online supplementary figure E6C), IL-18BPa (see online supplementary figure E6D) and their IL-1β/ILRA (see online supplementary figure E6E) and IL-18/IL-18BPa (see online supplementary figure E6F) ratios and IL-37 between the two group of subjects. Finally, BAL levels of IL-33 were under the detection limit of the assay (data not shown) in all subjects (see also online supplementary data repository).

### Correlations between inflammatory cell counts, IL-27-related molecules in the bronchi and clinical parameters

**Figure 4:**
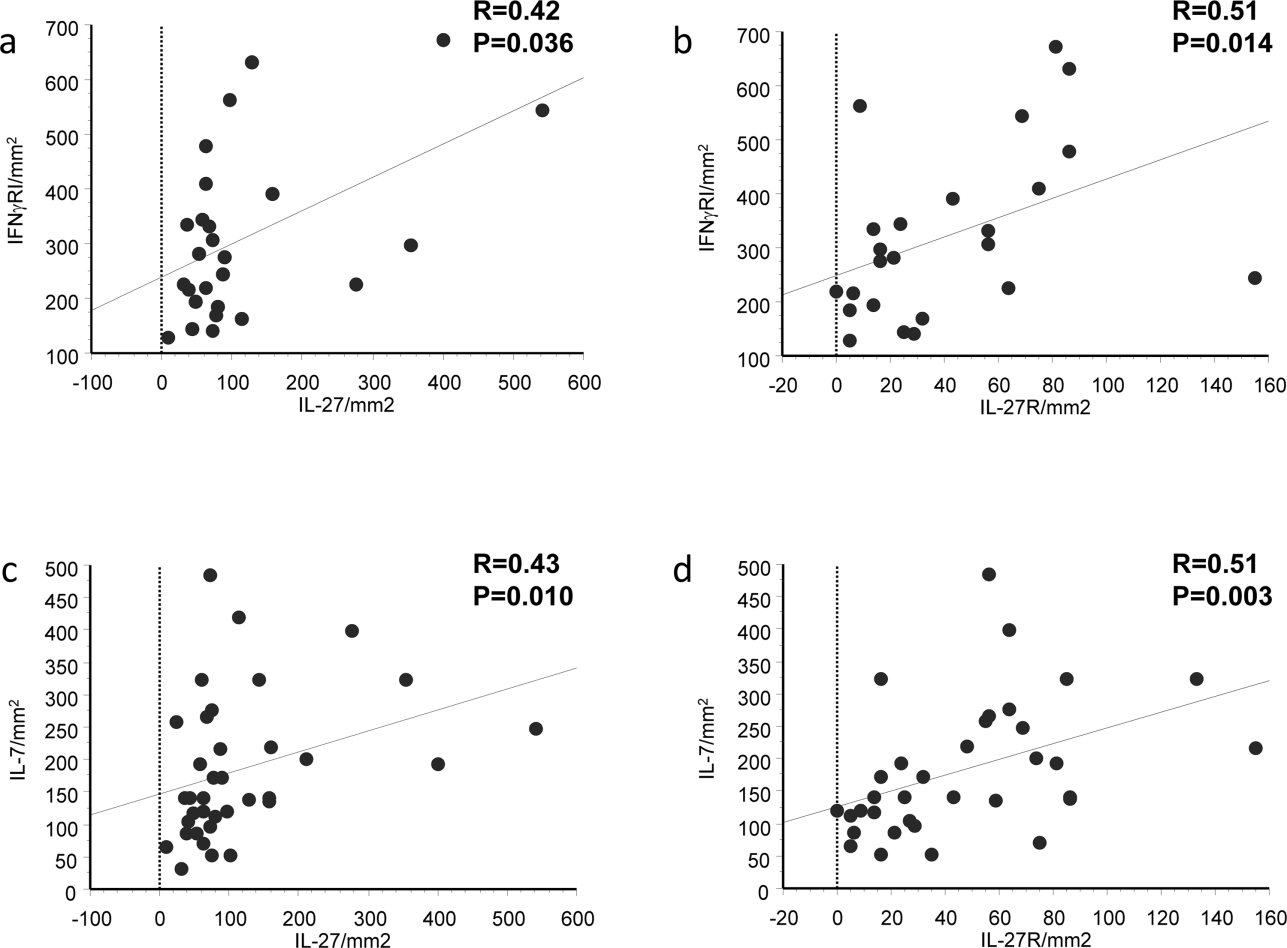
Regression analysis between numbers of (A) interferon (IFN)-γRI+ and interleukin (IL)-27+, (B) IFNγRI+ and IL-27R+, (C) IL-7+ and (D) IL-27+, IL-7+ and IL-27R+ cells in the bronchial submucosa of all patients with COPD. Correlation coefficients were calculated using the Spearman rank method.

Correlations restricted to patients with COPD alone show that the number of IFNγRI+ cells correlated with the number of IL-27+ (R=0.42, p=0.036) and IL-27R+ (R=0.51, p=0.014) cells in the bronchial submucosa (figure 4A,B). The number of IFNγRI+ cells also correlated with numbers of IL-7Rα (R=0.68, p=0.0009). IL-7+ cell numbers correlated with the number of IL-27+ (R=0.43, p=0.010) and IL-27R+ (R=0.51, p=0.003) cells in the bronchial submucosa of patients with stable COPD alone (figure 4C,D; see also online data supplement).

### Inflammation and oxidative stress enhance IL-27 mRNA and protein expression in NHBE cells in vitro, but not inflammasome-related mRNAs

Stimulation of human bronchial epithelial cells in vitro with a combination of oxidants (H_2_O_2_) and inflammatory cytokines significantly increased IL-27B mRNA and protein, but not the inflammasome-related IL-1β, IL-18 and caspase 1 encoding mRNAs (see online supplementary data repository and figures E7, E8 and E9).

## Discussion

We show significantly increased expression of IL-27 in the bronchial epithelium of patients with severe stable COPD compared with smoking and non-smoking controls. In addition, the number of IL-27+ and pSTAT1+ cells is also increased in the bronchial submucosa of patients with severe stable COPD compared with control smokers. This is associated with an increase in the number of IL-27R+ cells in patients with severe stable COPD compared with controls. Finally, in smokers, and in patients with COPD, the number of IFNγRI+ cells correlated with the number of IL-27+ and IL-27R+ cells. We failed to show evidence for NRLP3 activation in the airways of patients with stable COPD. Indeed, the levels of NRLP7 and IL-37, inhibitors of NLRP3 activity, were upregulated in COPD.

We have previously reported increased pSTAT4 expression linked to IFNγ expression^21^ but IL-27 can also induce IFNγ transcription through a STAT1- and STAT3-mediated process.^6^ IL-27 also increases proliferation and IFNγ and granzyme B production from human CD3-activated naïve CD8 cells. This results in increased CD8+ T-cell-mediated cytotoxicity.^22^ IL-27 also drives inducible regulatory T cells to produce IL-10.^23^ Although we observed a significant increase in the number of IL-10+ cells in bronchial submucosa of patients with COPD and healthy smokers, these numbers were very low in all subjects. Furthermore, there was no difference in IL-10R expression in the bronchial mucosa of patients with COPD. This, in conjunction with the previous lack of changes in FoxP3+ regulatory T cells^24^ and low FoxP3 expressions in BAL T lymphocytes from patients with stable COPD,^25^ suggests that a predominantly non-regulatory CD25+ helper T-cell population is present in smokers and patients with stable COPD. However, IL-10 expression is reduced in the sputum of patients with stable COPD^26^ and additional studies are required to clarify this discrepancy.

IL-27B mRNA and protein expression was upregulated in normal primary human bronchial epithelial cells by combined oxidative and pro-inflammatory stimuli. Although elevated levels of IL-27 have been reported in COPD sputum^27^ there was no increase in BAL IL-27 levels in patients with mild to severe stable COPD in this study. This may reflect the different compartments sampled by BAL and by sputum^28^ or that IL-27 is released from dead or dying sputum macrophages since we show here that CD68 cells are the main source of IL-27.

IL-7+ cell numbers were increased in the bronchial mucosa of stable COPD and control smokers compared with control non-smokers despite no changes in BAL IL-7 being found. IL-7 is produced by stromal and epithelial cells whereas its receptor is expressed mainly on T cells and monocytes.^7^ ^8^ The increased levels of IL-7, IL-7R and TSLPR observed here indicate that IL-7 may have a local pro-inflammatory function increasing the activation and survival of T cells and monocytes in the bronchial submucosa of patients with severe stable COPD.^29^

IL-7+ cell numbers correlate with IL-27+ and IL-27R+ cells, suggesting a functional relationship. The correlation between numbers of IL-7Rα+ and INFγRI on one side, and the inverse relationship between IL-7Rα and FEV_1_ % predicted on the other, suggests a link between increased expression of this receptor and increased severity in stable COPD. Overexpression of IL-7Rα is linked to increased severity of inflammatory bowel disease.^8^

High levels of IL-6 are released by sputum and BAL macrophages in vitro^3^ and IL-6 is increased in sputum of patients with COPD during exacerbations.^30^ In our study, BAL levels of IL-6, but not its soluble decoy receptor, were increased in COPD despite no change in the numbers of IL-6+ and IL-6R+ cells. Thus, our BAL data may simply reflect the presence of activated macrophages in the peripheral airways of patients with stable COPD. Soluble gp130 inhibits IL-6 trans-signalling via the sIL-6R and classic signalling via the mIL-6R.^31^ In animal models, sIL-6R-mediated signalling is an important intermediary in the resolution of neutrophilic inflammation,^31^ but we were unable to observe any significant difference in the expression of sgp130 in BAL from patients with stable COPD compared with control smokers with normal lung function.

**Figure 5:**
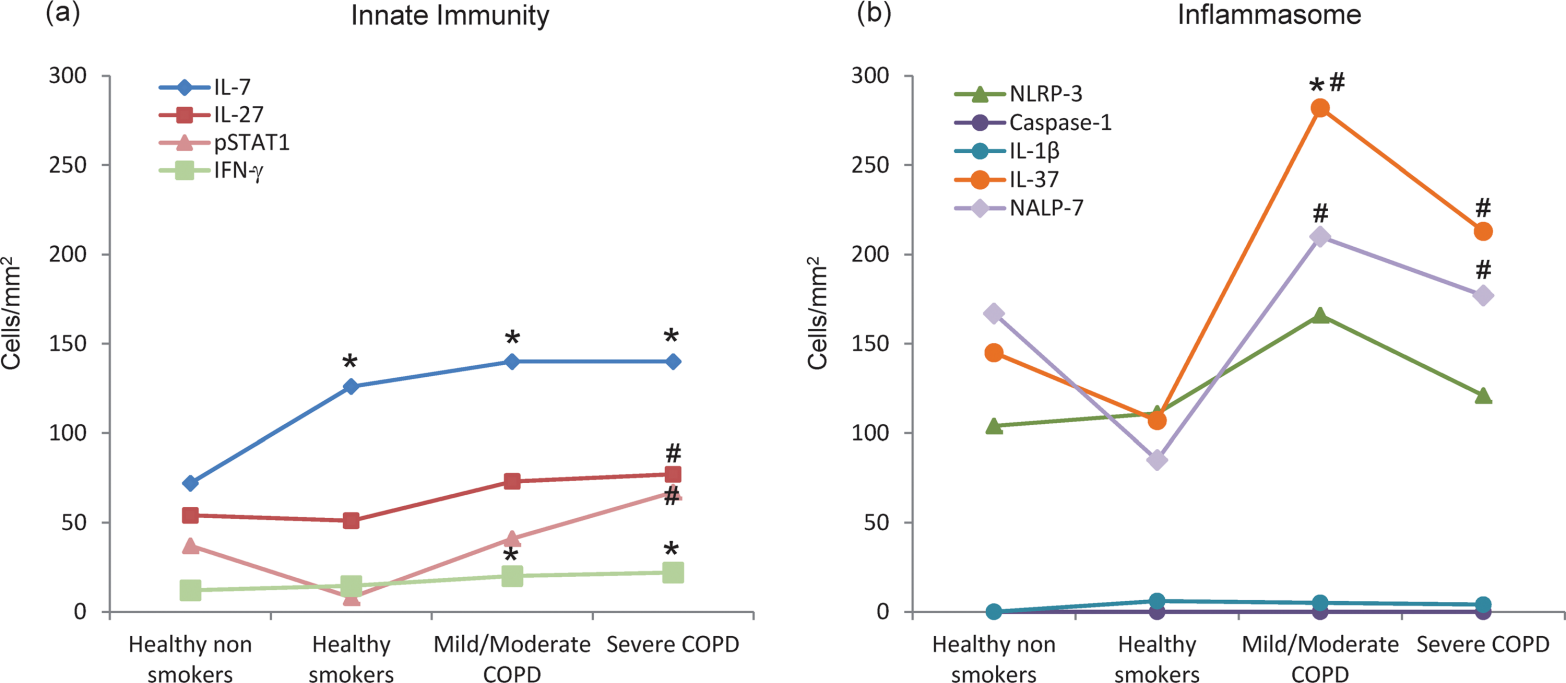
Schematic representation of the molecular variations related to innate immunity (A) and inflammasome (B) in the bronchial biopsy submucosa (values expressed as number of cells/mm^2^) of healthy non-smokers, healthy smokers, patients with mild/moderate stable COPD and patients with severe/very severe stable COPD. *Significantly different from healthy non-smokers; #significantly different from healthy smokers. With worsening of the disease, increased levels of interferon γ (IFNγ) are accompanied by increased levels of pSTAT1 and molecules inducing innate immunity such as interleukin (IL)-27 and IL-7 (A). Molecules inducing activation of the inflammasome are not changed (NLRP3, caspase-1) and IL-1β. Concomitantly, molecules inhibiting the inflammasome activation, such as IL-37 and NALP-7, are increased (B).

We report similar expression levels of NLRP3 in COPD and control subjects, an absence of caspase-1 expression in patients with COPD and controls, and no differences in the expression of IL-1β, its receptors and IL-18 in COPD bronchial biopsies and in the BAL level of IL-1β, IL-1RA, IL-18 and IL-18BPa, and their IL-1β/IL-1RA and IL-18/IL-18BPa ratios in patients with stable mild/moderate COPD versus control smokers with normal lung function. This suggests that the NRLP3 inflammasome only plays, at most, a minor role in the inflammatory response in the central airways of stable COPD. The increased levels of IL-18Rβ in bronchial biopsies of patients with severe COPD, compared with patients with mild COPD and control smokers, reported here, in the absence of a parallel increase in its own ligand, IL-18, may have a limited biological function (table 3, figure 5).

We did not find any difference in either IL-1β or IL-18 expression in the bronchial mucosa of patients with stable COPD and control subjects in this study. This is in contrast to increased levels of serum, sputum and BAL IL-1β,^32–35^ and plasma and sputum IL-18^36^ ^37^ reported in patients with COPD compared with control smokers and non-smokers. Furthermore, there is discordance between the levels of IL-18 and IL-1β in patients with COPD, suggesting that coordinated induction through the NRLP3 inflammasome is not critical for their expression.^36^ ^38^ Animal models of COPD also provide discordant data on the role of the NLRP3 inflammasome.^35^ ^38^ ^39^ Original data^38^ suggested a role for IL-1β in smoke-induced emphysema and airway remodelling but more recent data in mice favour a role for inflammasome-independent induction of IL-1β in driving smoke-induced inflammation.^35^ ^39^ The expression of other inflammasome components in patients with stable COPD has not been reported but the failure to detect increased IL-1β and IL-18 expression in the bronchial mucosa and BAL in this study suggests they may also play a limited role in patients with stable COPD. However, NRLP3 inflammasome activation may be important during viral and bacterial infections.^11^ ^13^ Previous studies have shown elevated levels of IL-1β in exhaled breath condensate and sputum during COPD exacerbations, particularly when associated with bacterial infections.^40^ We observed a modest, albeit significant, reduction of IL-1RA in the BAL of patients with stable COPD compared with control smokers with normal lung function, which is in line with previous studies,^41^ but without significant differences in their IL-1β/IL-1RA ratio. This suggests that the BAL decrease of this endogenous counter-regulatory mechanism of the IL-1β activation pathway may be clinically irrelevant. This is in line with data from unpublished clinical trials showing that monoclonal antibodies neutralising IL-1β signalling are ineffective in the treatment of patients with stable COPD (http://clinicaltrials.gov/ct2/show/results/NCT00581945).

Plasma and sputum HMGB1 levels have been previously reported to be increased in patients with stable COPD compared with control subjects^42^ but these studies were limited by not having controls matched for age and smoking history. BAL levels of HMGB1 have been reported as either increased in patients with stable COPD^41^ or no different from controls.^43^ In steroid-naïve patients with stable COPD, we found significantly decreased BAL HMGB1 levels, in comparison with age-matched control smokers with normal lung function, although this difference was lost when outliers were removed, again suggesting a minor role of inflammasome activation in the peripheral airways or a role limited to a subset of COPD patients only.

Overexpression of human IL-37 in mice results in downregulation of inflammation.^17^ The increased expression of IL-37 seen in the bronchial submucosa, but not in BAL, of patients with stable COPD compared with control smokers suggests a counter-regulatory role of this molecule. NALP7 attenuates caspase-1-dependent IL-1β secretion by inhibiting the processing of pro-IL-1β and pro-caspase 1.^12^ The increased expression of NALP7 in the submucosa, but not BAL, of patients with stable COPD may act to prevent the activation of the inflammasome pathway (figure 5).

One limitation of this study is the failure to apply a Bonferroni correction to the large number of biomarkers analysed. With Bonferroni correction the expression of IFNγRI, NALP7, STAT1, IL27R and IL7Rα in the submucosa and of NALP7 and TSLPR in the epithelium remain significantly different in patients with COPD, which supports the general message reported here.

In summary, our results showing an increased expression of the innate immunity cytokine IL-27 in the bronchial mucosa of patients with stable COPD indicate its potential role in the progression of severity of chronic bronchial inflammation. The absence of NRLP3 inflammasome increase in patients with COPD is associated with an increased expression of the inflammatory and inflammasome inhibitory molecules IL-37 and NALP7. These data suggest a relevant role for innate immunity and a silent state of inflammasome activation in patients with stable COPD.

## Supplementary Material

Web supplement
