# Supplementary material for: Innate immunity but not NLRP3 inflammasome activation correlates with severity of stable COPD
Source: Thorax. 2014 Jan 15;69(6):516–24. doi: 10.1136/thoraxjnl-2012-203062 (PMC4219154; doi:10.1136/thoraxjnl-2012-203062)
Supplement: Web supplement [file thoraxjnl-2012-203062-s1.pdf]

Thorax-2012-203062R2 online data repository

Online data repository

Innate immunity but not NLRP3 inflammasome activation correlates with severity of stable COPD

Antonino Di Stefano, PhD[1]*, Gaetano Caramori, MD[2]*, Adam Barczyk,[3] Chiara Vicari, BS[1], Paola Brun, PhD[4], Andrea Zanini, MD[1], Francesco Cappello, MD[5,6,7], Elvira Garofano, BS[2], Anna Padovani, BS[2], Marco Contoli, MD[2], Paolo Casolari PhD[2], Andrew L Durham[8], Kian Fan Chung[8], Peter J Barnes[8], Alberto Papi, MD[2], Ian Adcock, PhD[8]*, Bruno Balbi, MD[1]*.

Methods

Subjects

Subjects were recruited from the Respiratory Medicine Unit of the "Fondazione Salvatore Maugeri" (Veruno, Italy), the Section of Respiratory Diseases of the University Hospital of Ferrara, Italy and the Section of Respiratory Diseases of the University Hospital of Katowice, Poland for immunohistochemistry and ELISA experiments. The severity of the airflow obstruction was staged using GOLD criteria [www.goldcopd.org]. All former smokers had stopped smoking for at least one year. COPD and chronic bronchitis were defined, according to international guidelines, as follows: COPD, presence of a post-bronchodilator forced expiratory volume in one second (FEV_1)/forced vital capacity (FVC) ratio <70%; chronic bronchitis, presence of cough and sputum production for at least 3 months in each of two consecutive years (E1). All COPD patients were stable with no previous exacerbation in the six months before bronchoscopy. None of the subjects was treated with theophylline, antibiotics, antioxidants, mucolytics, and/or glucocorticoids in the month prior bronchoscopy. COPD patients were using short-acting inhaled β_2-agonists (SABA) or short-acting inhaled antimuscarinics (SAMA) prn or regular long-acting inhaled β_2-agonists (LABA) and/or regular inhaled anticholinergics, including SAMA or long-acting inhaled antimuscarinics (LAMA) at the dosage recommended in current COPD guidelines (www.goldcopd.org) at the time of their recruitment. The study conformed to the Declaration of Helsinki and was approved by the ethics committees of the Fondazione Salvatore Maugeri [Veruno (Novara), Italy], the University Hospital of Ferrara, Italy and the University Hospital of Katowice, Poland. Written informed consent was obtained from each subject and bronchial biopsies and BAL were performed according to the local ethic committee guidelines.

We obtained and studied bronchial biopsies from 55 subjects: 32 had a diagnosis of COPD in a stable clinical state (E2), 12 were current or ex-smokers with normal lung function, and 11 were non-smokers with normal lung function (Table 1 of the main manuscript). Two out of 12 current or ex-smokers were pure volunteers. All the other control subjects, smokers, ex-smokers and non-smokers, attended our respiratory clinic mostly for of unexplained persistent cough. At their first visit they were screened and after exclusion of the inappropriate subjects determined by the clinical history, physical examination, lung function and imaging studies and laboratory examinations for lung, upper airways and/or cardiac or systemic disease, subjects were recruited. COPD patients were divided in two groups, according to the grade of severity of their airflow limitation (mild/moderate, grade I-II; or severe/very severe, grade III-IV; n=14 and 18, respectively) (E1). Subjects in all four groups were age-matched. The smoking history was similar in the three smoker groups: mild/moderate and severe/very severe COPD, and healthy smokers with normal lung

function. Values of FEV_1 (% predicted) and FEV_1/FVC (%) were significantly different in the groups with mild/moderate and severe/very severe COPD compared to both control groups (healthy smokers and healthy non-smokers). Severe/very severe COPD patients also differed significantly from mild/moderate COPD patients (for overall groups, ANOVA test: $p<0.0001$ for FEV_1% predicted and FEV_1/FVC% values). Forty-three percent (n=14) of the total COPD patients and 41% (n=5) of healthy smokers with normal lung function also had symptoms of chronic bronchitis. There was no significant difference when COPD patients and healthy smokers were compared for the presence of chronic bronchitis.

The clinical details of the subjects undergoing bronchoalveolar lavage (BAL) are summarized in Table 2 of the main manuscript. We analyzed the BAL fluid obtained from 26 COPD and 18 control smokers with normal lung function but due to the necessity to concentrate the BAL supernatants for many ELISA assays we were unable to perform all these assays in all subjects. The results provided for each ELISA are the data from 15 COPD and 14 control smokers with normal lung function.

Of the 44 subjects included for the bronchoalveolar lavage (BAL) analysis, 21 patients were recruited in the Veruno's Hospital and 23 subjects in Poland and Ferrara. All the BAL procedures were well standardized in accordance with standard guidelines. All the bronchoscopists in the three centers followed the same SOP for BAL collection and processing and the ELISAs on the BAL supernatants were run in a single center (Ferrara).

Lung function tests and volumes

Pulmonary function tests were performed as previously described (E2) according to published guidelines (E3). Pulmonary function tests included measurements of FEV_1 and FEV_1/FVC under baseline conditions in all the subjects examined (6200 Autobox Pulmonary Function Laboratory; Sensormedics Corp., Yorba Linda, CA). In order to assess the reversibility of airflow obstruction and post bronchodilator functional values the FEV_1 and FEV_1/FVC% measurements in the groups of subjects with $FEV_1/FVC\% \leq 70\%$ pre-bronchodilator was repeated 20 min after the inhalation of 0.4 mg of albuterol.

Fiberoptic bronchoscopy, collection and processing of bronchial biopsies

Subjects were at the bronchoscopy suite at 8.30 AM after having fasted from midnight and were pre-treated with atropine (0.6 mg IV) and midazolam (5-10 mg IV). Oxygen (3 l/min) was administered via nasal prongs throughout the procedure and oxygen saturation was monitored with a digital oximeter. Using local anaesthesia with lidocaine (4%) to the upper airways and larynx, a fiberoptic bronchoscope (Olympus BF10 Key-Med, Southend, UK) was passed through the nasal

passages into the trachea. Further lidocaine (2%) was sprayed into the lower airways, and four bronchial biopsy specimens were taken from segmental and subsegmental airways of the right lower and upper lobes using size 19 cupped forceps. Bronchial biopsies for immunohistochemistry were extracted from the forceps and processed for light microscopy as previously described (E2). Two samples were embedded in Tissue Tek II OCT (Miles Scientific, Naperville, IL), frozen within 15 min in isopentane pre-cooled in liquid nitrogen, and stored at –80°C. The best frozen sample was then oriented and 6μm thick cryostat sections were cut for immunohistochemical light microscopy analysis and processed as described below.

Immunohistochemistry

Two sections from each sample were stained applying immunohistochemical methods with a panel of antibodies specific for inflammatory cells, innate immune mediators and inflammasome components (Table E1). Briefly, after blocking non-specific binding sites with serum derived from the same animal species as the secondary antibody, primary antibody was applied at optimal dilutions in TRIS-buffered saline (0.15M saline containing 0.05 M TRIS-hydrochloric acid at pH 7.6) and incubated for 1hr at room temperature in a humid chamber. Antibody binding was demonstrated with secondary antibodies anti-mouse (Vector, BA 2000), anti-rabbit (Vector, BA 1000) or anti-goat (Vector, BA 5000) followed by ABC kit AP AK5000, Vectastain and fast-red substrate (red color) or ABC kit HRP Elite, PK6100, Vectastain and diaminobenzidine substrate (brown color). Slides were included in each staining run using human tonsil, nasal polyp or breast cancer, as a positive control. For the negative control slides, normal non-specific goat, mouse or rabbit immunoglobulins (Santa Cruz Biotechnology, Santa Cruz, CA, USA) were used at the same protein concentration as the primary antibody.

Scoring system for immunohistochemistry

Morphometric measurements were performed with a light microscope (Leitz Biomed, Leica Cambridge, UK) connected to a video recorder linked to a computerized image system (Quantimet 500 Image Processing and Analysis System, Software Qwin V0200B, Leica). Light-microscopic analysis was performed at a magnification of 630x.

The immunostaining for all the antigens studied was scored (range: 0 = absence of immunostaining to 3 = extensive intense immunostaining) in the intact (columnar and basal epithelial cells) bronchial epithelium, as previously described (E2). The final result was expressed as the average of all scored fields performed in each biopsy. A mean±SD of 0.70±0.26 millimeters of epithelium was analyzed in COPD patients and control subjects.

Immunostained cells in bronchial submucosa (lamina propria) were quantified 100 μm beneath the epithelial basement membrane in several non-overlapping high-power fields until the whole specimen was examined. The final result, expressed as the number of positive cells per square millimeter, was calculated as the average of all the cellular counts performed in each biopsy.

Double staining and confocal microscopy

Four patients with COPD (FEV_1, 64±15%; FEV_1/FVC, 61±8%) and 4 control smokers (FEV_1, 104±14%; FEV_1/FVC, 81±3%) were used for double staining immunofluorescence and confocal microscopy. Double staining was performed as previously reported (E2). For confocal microscopy sections were fixed with 4% paraformaldehyde, washed with phosphate buffered saline (PBS) and incubated (1 hour) with PBS containing 5% bovine serum albumin and 5% donkey serum. After blocking, sections were incubated 1 hour with the primary antibodies diluted as indicated in Table E1 in PBS containing 5% bovine serum albumin. The following antibodies were used: rabbit anti-human IL-27 (LS B2565, R&D); and mouse anti human CD4, CD8 and CD68, M716, M7103 and M814, respectively, Dako). After washing with PBS, the preparations were incubated for a further 30 min with the appropriate secondary Alexa Fluor 488- or Alexa Fluor 647 conjugated antibodies diluted 1:200 in PBS. Negative controls included non-specific mouse and rabbit immunoglobulins revealed as for primary antibodies. Slides were mounted using a specific mounting medium (Vector, H-1400, Vectashield Hard Set). The slides for confocal microscopy were analysed using a three-channel Leica TCS SP5 laser scanning confocal microscope. The Leica LCS software package was used for acquisition, storage, and visualization. The quantitative estimation of co-localized proteins was performed calculating the "co-localization coefficients" (E4, E5).

Bronchoalveolar lavage

Bronchoalveolar lavage (BAL) was performed from the right middle lobe using four successive aliquots of 50ml of 0.9% NaCl. BAL cells were spun (500×*g*; 10min) and washed twice with Hanks' buffered salt solution (HBSS). Cytospins were prepared and stained with May-Grünwald stain for differential cell counts. Cell viability was assessed using the trypan blue exclusion method. BAL supernatants were aliquoted and left at -80°C before its use for the ELISA assays summarised in Table E2. These assays have been performed according to the manufacturers' instructions.

Cell culture and treatments

We used normal human bronchial epithelial cells (NHBE), of non-smoking subjects obtained from Lonza (Cologne, Germany) grown in BEGM media (Lonza) with Singlequot supplement (Lonza) following the suppliers instructions. Cells were passaged using the ReagentPack ™ Subculture Reagents (Lonza) following the manufacturer's instructions. Passage number of cells used in this study ranged from 3 to 6. Prior to all the experiments, 70-80% cell monolayers were incubated in supplement–free medium for the 18hrs. The cells were treated with hydrogen peroxide (H_2O_2, 100μM), IL-1β (1 ng/ml), combined IL-1β and H_2O_2, cytomix (50ng/ml of TNFα, IL-1β and IFNγ) or combined cytomix and H_2O_2 for 2hrs to analyse the messenger RNAs or 24 hours to measure protein expression. All experiments were performed at least three times.

Extraction and quantification of RNA and qRT-PCR from NHBE

Total RNA was isolated from cells using the RNeasy RNA extraction kit following manufacturer's instructions (Qiagen, UK). cDNA was made from quantified RNA by reverse transcription using the high capacity cDNA kit following manufacturer's instructions (Applied Biosystems, UK). The expression of genes of interest was measured using Syber green (Qiagen, UK) for qPCR in a Corbett Rotorgene 6 (Corbett, Cambridge, UK). We detected the expression of IL-27p28 and EBI3 (IL-27B) using the following primers (E6): IL-27p28 forward, agc tgc atc ctc tcc atg tt; reverse, gag cag ctc cct gat gtt tc; EBI3 (IL-27B) forward, tgt tct cca tgg ctc cct ac; reverse, gct ccc tga cgc ttg taa c. mRNA was normalized using a housekeeping gene 18S, using the following primers 18S forward ctt aga ggg aca agt ggc g; reverse acg ctg agc cag tca gtg ta, for each experimental condition.

Extraction and quantification of IL-27 protein from NHBE

IL-27 levels in the cell culture supernatant following stimulation with H_2O_2 (100μM), IL-1β (1 ng/ml), combined IL-1β and H_2O_2, cytomix (50ng/ml of TNFα, IL-1β and IFNγ) or combined cytomix and H_2O_2 for 24 hours was quantified by sandwich ELISA (R&D Systems Europe, Abingdon, UK) exactly according to the manufacturer`s instructions.

Statistical analysis

Group data were expressed as mean (standard deviation) for functional data or median (range) or interquartile range (IQR) for morphologic data. We tested for a normal distribution for functional data (i.e. FEV1%, FVC, age etc.) and for a non normal distribution for morphological parameters. Normality tests were performed on all group data. The Grubb's outlier test was used to determine

whether individual values were outside the rest of the group. Then we applied the analysis of variance (ANOVA) for functional data in comparing subgroups of patients and control subjects for functional data. The non parametric Kruskal Wallis test was applied for multiple comparisons, without application of Bonferroni correction, when morphologic data were analysed followed by the Mann-Whitney U test for comparison between groups. The statistical guide to GraphPad Prism recommends that the Bonferroni correction should not be used when comparing more than 5 variables due to the conservative nature of the test and the subsequent likeliness of missing real differences. We believe that this comparative analysis is of value and represents part of our informative findings. For this reason we applied specific non parametric statistical tests to our data of Table 3 without including the Bonferroni correction. To verify the degree of association between functional or morphological parameters, in all smokers with and without COPD or in smokers with COPD alone the correlation coefficients between functional and morphological and morphological-morphological data were calculated using the Spearman rank method. Probability values of $p<0.05$ were considered significant. Data analysis was performed using the Stat View SE Graphics program (Abacus Concepts Inc., Berkeley, CA-USA) and GraphPad Prism software (www.graphpad.com/scientific-software/prism/).

Results

Measurement of inflammatory cells in the bronchial submucosa

The results are summarized in Table E3. The number of CD8 positive lymphocytes was significantly increased in severe/very severe (p=0.021) and mild/moderate (p=0.027) stable COPD compared to control non-smokers. The number of CD4 positive lymphocytes did not differ significantly between the four groups of subjects. Compared with control non-smokers, the number of CD68 positive macrophages was significantly higher in severe/very severe (p=0.033) and mild/moderate (p=0.036) stable COPD. The number of neutrophils was also significantly higher in severe/very severe stable COPD patients compared with control smokers (p=0.011) and non-smokers (p=0.010). Stable COPD patients with chronic bronchitis had a similar number of neutrophils when compared with stable COPD patients without chronic bronchitis (data not shown).

Immunohistochemistry for innate immunity and inflammasome pathways in the bronchial submucosa

For all the proteins studied mononuclear cells (lymphocytes and macrophages) and endothelial cells were the most represented immunostained cells in the submucosa. The number of IFNγ+ cells was significantly higher in mild/moderate (p=0.010) and severe (p=0.008) COPD compared to control non-smokers, confirming previously reported data (E2). The number of IFNγRI+ cells was also increased in severe COPD compared to mild COPD (p=0.031), control smokers (p=0.0035) and control non-smokers (p=0.006). IL-18Rβ showed a slight increase in severe COPD compared to mild/moderate COPD (p=0.045) and control smokers (p=0.039) but did not differ in comparison with control non-smokers. The number of IL-7+ (Figure E1) cells was higher in severe (p=0.008), mild/moderate COPD (p=0.010) and in control smokers (p=0.012) compared to control non-smokers. In addition the number of IL-7Rα+ cells was significantly higher in severe compared to mild/moderate COPD (p=0.040), control smokers (p=0.009) and control non-smokers (p=0.002). IL-10 was poorly expressed but number of IL-10+ cells was higher in severe (p=0.005), mild/moderate COPD (p=0.047) and in control smokers (p=0.054) in comparison with control non-smokers.

The number of IL-27+ (Figure 1) as well as pSTAT1+ (Figure E2) cells was significantly higher in severe COPD (p=0.032 and p=0.018, respectively) compared to control smokers but did not differ in comparison with the other groups. Interestingly, compared to control smokers and non-smokers, the number of IL-27R+ cells was higher in severe (p=0.010 and p=0.002, respectively) and mild/moderate COPD (p=0.054 and p=0.009, respectively). Similarly, the number of total STAT1+

cells was significantly higher in severe (p=0.0043 and p=0.015, respectively) and mild/moderate COPD (p=0.022 and p=0.029, respectively).

No significant differences were observed for IL-1β, IL-1βRI, IL-1βRII, caspase-1, IL-18, IL-18Rα, IL-18BP, NLRP3, IL-6, IL-6Rα, IL-10Rα, IL-33, ST-2 and TSLP immunostaining in the four groups of subjects examined. However, caspase-1 staining was observed in the positive control represented by human breast tumour tissue (Table E1).

Double staining and confocal microscopy

Double staining for identification of CD4+, CD8+ lymphocytes and macrophages (CD68+) co-expressing IL-27 was performed in four representative healthy smokers with normal lung function and in four patients with COPD. We found no difference in the percentage (mean±SD) of CD4+IL-27+ double stained cells between control smokers (12±2%) and COPD patients (11±6%). Similarly, there was no difference in the percentage of CD8+IL-27+ double stained cells between control smokers (14±11%) and COPD patients (9±2%). Interestingly, the percentage of CD68+IL-27+ double stained cells was significantly increased in COPD patients (34±8%) compared to control smokers (8±2%, p=0.0209) (Figure E4).

ELISA assays in the BAL supernatants

The BAL levels of IL-6 were significantly increased in stable COPD patients compared to the control smokers with normal lung function [median (range) 99.4(54.8-489.6) pg/ml vs 36.6 (28.2-76-8) pg/ml, respectively; p=0.0001; Figure 3A), without a significant change in sIL-6R expression between the two groups [127.2(28-435-7) pg/ml vs 197.1(21.7-843-2) pg/ml, respectively; p=0.2658; Figure E5A].

The BAL levels of IL-7 [9.4(5-35.9) pg/ml vs 9.1(2.5-99.7) pg/ml, respectively; p=0.8273; Figure E5B], IL-27 [4.3(0.3-64.6) pg/ml vs 15.1(0.8-248.1) pg/ml, respectively; p=0.112; Figure E5C] and sgp130 [3213(278.4-9350) pg/ml vs 5663(453.2-11660) pg/ml, respectively; p=0.2850; Figure E6A] were not significantly different between the two group of subjects. However, the BAL level of HMGB1 was significantly decreased in stable COPD patients compared to control smokers with normal lung function [2340 (314-3550) pg/ml vs 5105 (0-103521) pg/ml, COPD vs control smokers respectively; p=0.0174; Figure 3C]. However, this difference was lost after removal of the outliers.

The BAL level of soluble ST2 [55(30.3-563) pg/ml vs 168.5(57-1770) pg/ml, respectively; p=0.0073; Figure 3D] and IL-1RA [147(26.7-390) pg/ml vs 333(53.4-1707) pg/ml, respectively; p=0.0307; Figure 3B] are both significantly decreased in stable COPD patients compared to control smokers with normal lung function without significant changes in the BAL level of IL-1β

[0.22(0.11-1.2) pg/ml vs 0.4(0.13-6.7) pg/ml, respectively; p=0.2214; Figure E6B], IL-18 [13.1(4.7-82.2) pg/ml vs 18(8.4-73.2) pg/ml, COPD vs control smokers respectively; p=0.1063; Figure E6C] and IL-18BPa [78.9(25.2-102.8) pg/ml vs 57.9(35.2-558.4) pg/ml, COPD vs control smokers respectively; p=0.3947; Figure E6D]. In addition, the IL-1β/ILRA [0.002(0.0007-0.007) vs 0.001 (0.0002-0.02), COPD vs control smokers respectively; p=0.6625; Figure E6E] and IL-18/IL-18BPa [0.2(0.06-1) vs 0.3 (0.09-2), COPD vs control smokers respectively; p=0.3710; Figure E6F] ratios and IL-37 levels [60(53.4-74.1) pg/ml vs 57.8(44.4-68.1) pg/ml, COPD vs control smokers respectively; p=0.1980; Figure E5D] were not different between the two group of subjects. The BAL level of IL-33 was under the detection limit of the assay (data not shown) in all subjects.

Correlations between inflammatory cell counts, IL-27 related molecules in the bronchi and clinical parameters

In all smokers there was a positive correlation between the number of IFNγRI+ cells and the number of IL-27+ (R=0.43, p=0.009) and IL-27R+ (R=0.56, p=0.001) cells in the bronchial submucosa. The number of IFNγRI+ cells also correlated with numbers of IL-7Rα+ cells (R=0.62, p=0.0003). Numbers of IL-7Rα+ cells correlated inversely with pre- and post-bronchodilator FEV_1% predicted values (R=-041, p=0.005 and R=-0.42, p=0.013, respectively). Furthermore, the number of IL-7+ cells correlates significantly with the number of IL-27+ (R=0.49, p=0.0007) and IL-27R+ (R=0.43, p=0.003) cells in the bronchial submucosa of all smokers.

When correlations were restricted to patients with COPD alone all reported correlations were maintained. In fact, the number of IFNγRI+ cells correlated again with the number of IL-27+ (R=0.42, p=0.036) and IL-27R+ (R=0.51, p=0.014) cells in the bronchial submucosa (Figure 4, panels a and b). The number of IFNγRI+ cells also correlated with numbers of IL-7Rα (R=0.68, p=0.0009).

Numbers of IL-7Rα correlated inversely with pre- and post-bronchodilator FEV1% predicted values (R=-045, p=0.009 and R=-0.36, p=0.049, respectively). Furthermore, the number of IL-7+ cells correlated significantly with the number of IL-27+ (R=0.43, p=0.010) and IL-27R+ (R=0.51, p=0.003) cells in the bronchial submucosa of COPD patients alone (Figure 4, panels c and d).

No other significant correlations were found between inflammatory cells, innate immunity and inflammasome pathways expression in bronchial mucosa and/or BAL and any clinical parameters.

IL-27 and inflammasome mRNAs expression in NHBE cells induced by oxidative and inflammatory stimuli "In vitro"

Normal human bronchial epithelial (NHBE) cells were stimulated with H_2O_2 (100μM), IL-1β (1ng/ml) and H_2O_2 + IL-1β at the same concentrations as for single treatments, cytomix alone (TNFα, IL-1β and IFNγ each at 50ng/ml) and combined cytomix + H_2O_2 at the same concentrations as for single treatments and quantified the expression of IL-27p28 (Figure E7A) and IL-27B (Figure E7B) mRNA by qRT-PCR. IL-27B mRNA was significantly increased (Figure E7B and E7C) after combined treatment with cytomix plus H_2O_2 (n=3, paired T test, $p<0.05$). The same stimulation did not significantly stimulate the expression of inflammasome-related IL-1β, IL-18 and caspase 1 encoding mRNAs (Figures E8A, E8B and E8C). The 2hr time point was selected after an initial time-course study was performed (data not shown).

IL-27 protein expression in NHBE cells induced by oxidative and inflammatory stimuli "In vitro"

The levels of IL-27 protein was measured in the supernatant and whole cell extract of NHBE cells, treated with H_2O_2 (100μM), IL-1β (1ng/ml), cytomix (50ng/ml TNFα, IL-1β and IFNγ) and combined IL-1β+H_2O_2 or cytomix+H_2O_2. No IL-27 protein was detected in the supernatant of any of the samples (data not shown). Protein from the whole cell extract showed a small but significant increase in the levels of IL-27 when treated with the combined treatment of cytomix+H_2O_2 s (n=3, paired T test, $p<0.05$) (Figure E9).

References

E1. Vestbo J, Hurd SS, Agusti AG, Jones PW, Vogelmeier C, Anzueto A, et al. Global Strategy for the Diagnosis, Management and Prevention of Chronic Obstructive Pulmonary Disease, GOLD Executive Summary. Am J Respir Crit Care Med. 2013;187:347-365.

E2. Di Stefano A, Caramori G, Gnemmi I, Contoli M, Bristot L, Capelli A, Ricciardolo FL, Magno F, D'Anna SE, Zanini A, Carbone M, Sabatini F, Usai C, Brun P, Chung KF, Barnes PJ, Papi A, Adcock IM, Balbi B. Association of increased CCL5 and CXCL7 chemokine expression with neutrophil activation in severe stable COPD. Thorax. 2009;64:968-975.

E3. Quanjer PH, Tammeling GJ, Cotes JE, Pedersen OF, Peslin R, Yernault JC. Lung volumes and forced ventilatory flows. Report Working Party Standardization of Lung Function Tests, European Community for Steel and Coal. Official Statement of the European Respiratory Society. Eur Respir J Suppl. 1993;16:5-40.

E4. Gonzalez R.C., Wintz P. Digital Image Processing, 2nd edn., Addison Wesley Publication Company, Mass. USA 1987.;Costes S.V., Daelemans D., Cho E.H., Dobbin Z., Pavlakis G., Lockett S. Automatic and quantitative measurement of protein-protein colocalization in live cells. Biophysical Journal 2004;86:3993-4000.

E5. Manders E.M.M., Verbeek F.J., Aten J.A. Measurement of co-localization of objects in dual-colour confocal images. J Microscopy 1993;169:375-438.

E6. Krumbiegel D, Anthogalidis-Voss C, Markus H, Zepp F, Meyer CU. Enhanced expression of IL-27 mRNA in human newborns. Pediatr Allergy Immunol. 2008;19:513-516.

Table E1. Primary antibodies and immunohistochemical conditions used for identification of cytokines, proteins and inflammatory cells

Target	Supplier	[a]Cat#	Source	Dilution	Positive control
**CD4**	Dako	M716	Mouse	1:100	Human tonsil
**CD8**	Dako	M7103	Mouse	1:200	Human tonsil
**CD68**	Dako	M814	Mouse	1:200	Human tonsil
**Neutrophil elastase**	Dako	M752	Mouse	1:100	Nasal polyp
**IL-6**	R&D	AF206NA	Goat	1:80	Human tonsil
**IL-6 Rα**	Diaclone	852.033.020	Mouse	1:20	Human tonsil
**IL-7**	R&D	MAB207	Mouse	1:40	Nasal polyp
**IL-7 Rα**	R&D	AF306PB	Goat	1:80	Nasal polyp
**IL-10**	R&D	AF217NA	Goat	1:100	Nasal polyp
**IL-10 Rα**	R&D	AF274NA	Goat	1:50	Nasal polyp
**IL-27p28**	R&D	LS B2565	Rabbit	1:250	Human tonsil
**IL-27 R**	R&D	ANP1619	Rabbit	1:300	Human tonsil
**IL-33**	S.Cruz	Sc-98659	Rabbit	1:150	Nasal polyp
**ST-2**	S.Cruz	Sc-18687	Goat	1:100	Nasal polyp
**TSLP**	Peprotech	500-P258	Rabbit	1:40	Nasal polyp
**TSLP-R**	R&D	AF981	Goat	1:25	Nasal polyp
**IL-18**	S.Cruz	Sc-133127	Mouse	1:50	Nasal polyp
**IL-18 Rα**	R&D	AF840	Goat	1:15	Nasal polyp
**IL-18 Rβ**	S.Cruz	Sc-107635	Goat	1:75	Nasal polyp
**IL-18 BP**	Epitomics	1893-1	Rabbit	1:100	Nasal polyp
**NLRP-3**	Sigma	C-33680	Rabbit	1:75	Nasal polyp
**Caspase-1**	Epitomics	3345-1	Rabbit	1:200	Breast Tumor
**IL-1β**	R&D	AB201NA	Goat	1:150	Human tonsil
**IL-1βRI**	S.Cruz	Sc-66054	Mouse	1:50	Human tonsil
**IL-1βRII**	R&D	AF263NA	Goat	1:50	Human tonsil
**IL-37**	R&D	AF1975	Goat	1:40	Nasal polyp
**NALP7 (NLRP7)**	Thermo Scientific	PA5-21023	Rabbit	1:400	Nasal polyp
**STAT1**	Atlas	HPA000931	Rabbit	1:200	Human tonsil
**pSTAT1**	BD	612133	Mouse	1:80	Human tonsil
**IFN-γ**	R&D	MAB2851	Mouse	1:100	Human tonsil
**IFN-γ RI**	R&D	AF673	Goat	1:30	Human tonsil

[a]Cat#, catalogue number

Table E2. Summary of the ELISA assays performed on BAL supernatants

Specificity	Molecular weight (kDa)	Manufacturer website	Catalogue code	Standard curve sensibility range	
IL-1β	17.5	www.rndsystems.com	HSLB00C	0-8 pg/ml	Not concentrated
IL-1RA	22-25	www.rndsystems.com	DRA00B	0-2000 pg/ml	Not concentrated
IL-6	20-30	www.raybiotech.com	ELH-IL6-001	0-1000 pg/ml	Concentrated x30 with Vivaspin
IL-6sR	50-55	www.biosupply.co.uk	EL10034	0-2000 pg/ml	Concentrated x30 with Vivaspin
sgp130	130	www.rndsystems.com	DGP00	0-8 ng/ml	Concentrated x30 with Vivaspin
IL-7	20-25	www.raybiotech.com	ELH-IL7-001	0-1000 pg/ml	Concentrated x30 with Vivaspin
IL-18	18-24	www.mblintl.com	7620	0-1000 pg/ml	Not concentrated
IL-18BPa	22-40	www.raybiotech.com	ELH-IL18BPA-001	0-18.000 pg/ml	Not concentrated
IL-27	69	www.uscnk.us	E90385Hu	0-1000 pg/ml	Concentrated x30 with Vivaspin
IL-33	30	www.biolegend.com	435907	0-1000 pg/ml	Concentrated x30 with Vivaspin
IL-37	30	www.adipogen.com	AG-45A-0041EK-KI01	0-1000 pg/ml	Not concentrated
ST2/IL-1R4	60	www.rndsystems.com	DST200	0-2000 pg/ml	Concentrated x30 with Vivaspin
HMGB1	30	www.ibl-international.com	ST51011	0-80 ng/ml	Not concentrated

Table E3. Immunohistochemical quantification of inflammatory cells in the bronchial mucosa

Target Group					
	**Control non-smokers**	**Control smokers normal lung function**	**COPD stage I/II (mild/moderate)**	**COPD stage III/IV (severe/very severe)**	***p* value**
**Submucosa (cells/mm^2)**					
**CD4**	168 (88-378)	218 (37-500)	245 (86-731)	252 (42-671)	0.360
**CD8**	120 (15-301)	187 (78-657)	208 (86-523) #	215 (59-355) #	0.125
**CD68**	284 (110-516)	369 (97-945)	566 (158-833) #	428 (204-1054) #	0.130
**Neutrophil Elastase**	89 (58-179)	101 (17-308)	125 (28-512)	173 (47-500) #ε	0.030

Abbreviations: COPD, chronic obstructive pulmonary disease; Data expressed as median (range). Statistics: Kruskal-Wallis test for multiple comparisons were performed and the "p" values across groups shown. For comparison between groups the Mann-Whitney U test was applied: $^{\#}p<0.05$, significantly different from control non-smokers; $^{\varepsilon}p<0.05$, significantly different from control smokers with normal lung function.

Data supplement E-Figures

Figure E1

Photomicrographs showing the bronchial mucosa from (a) control non-smoker, (b) control healthy smoker with normal lung function, (c) mild/moderate stable chronic obstructive pulmonary disease (COPD) and (d) severe stable COPD immunostained for identification of IL-7+ cells (arrows) in the bronchial submucosa. Results are representative of those from 11 non-smokers, 12 healthy smokers, 14 mild/moderate COPD and 18 with severe COPD. Bar=30 microns.

Figure E2

Photomicrographs showing the bronchial mucosa from (a) control non-smoker, (b) control healthy smoker with normal lung function, (c) mild/moderate stable chronic obstructive pulmonary disease (COPD) and (d) severe stable COPD immunostained for identification of pSTAT1+ cells (arrows) in the bronchial submucosa. Results are representative of those from 11 non-smokers, 12 healthy smokers, 14 mild/moderate COPD and 18 with severe COPD. Bar=30 microns.

Figure E3

Photomicrographs showing the bronchial mucosa from (a) control non-smoker, (b) control healthy smoker with normal lung function, (c) mild/moderate stable chronic obstructive pulmonary disease (COPD) and (d) severe stable COPD immunostained for identification of NALP7+ cells (arrows) in the bronchial epithelium and submucosa. Results are representative of those from 11 non-smokers, 12 healthy smokers, 14 mild/moderate COPD and 18 with severe COPD. Bar=30 microns.

Figure E4

Representative double-labelled confocal fluorescence images showing double staining for CD68+ macrophages and IL-27 in the bronchial mucosa from four healthy smokers with normal lung function (A-B) and four patients with stable chronic obstructive pulmonary disease (COPD) (C-D). Images A and C were obtained from one healthy control smoker and one patient with severe COPD, respectively. Arrows in panel C indicate double stained cells. Images B and D show the co-expression levels of IL-27 (Alexa Fluor 488-green) and CD68 (Alexa Fluor 647-red) and represent the correlation cytofluorogram of the images in A and C, respectively.

Figure E5

BAL supernatant levels of (A) IL-6R, (B) IL-7, (C) IL-27 and (D) IL-37 in stable COPD patients (n=15) compared to the control smokers with normal lung function (n=14). Exact p values are shown above each graph.

Figure E6

BAL supernatant levels of (A) sgp130, (B) IL-1β, (C) IL-18, (D) IL-18BPa and (respectively E and F) their IL-1β/IL.1RA and IL-18/IL-18BPa ratios in stable COPD patients (n=15) compared to the control smokers with normal lung function (n=14). Exact p values are shown above each graph.

Figure E7.

In vitro expression of IL-27p28 (A) and IL-27B (B and C) in normal primary human bronchial epithelial cells treated with H_2O_2 (100μM), IL-1β (1ng/ml), cytomix (50ng/ml TNFα, IL-1β and IFNγ) and combined IL-1β + H_2O_2 or cytomix + H_2O_2. Combined treatment with cytomix + H_2O_2 significantly up-regulated the mRNA expression of IL-27B (B and C) (n=3, paired T test, $p<0.05$). Panel C shows a direct comparison between results from 3 subjects to indicate level of variability.

Figure E8

In vitro expression of expression of the inflammasone-related genes (*IL1B*, *IL18* and *CASP1*) in normal human bronchial epithelial cells were measured in cells treated with H_2O_2 (100μM), IL-1β (1ng/ml), cytomix (50ng/ml TNFα, IL-1β and IFNγ) and combined IL-1β + H_2O_2 or cytomix + H_2O_2. Whilst treatment with cytomix alone did significantly increase *IL1B* gene expression (n=3, paired T test, $p<0.05$) no other gene or treatment showed significant increase in expression relative to the untreated controls.

Figure E9

In vitro expression of IL-27 protein in normal primary human bronchial epithelial cells treated with H_2O_2 (100μM), IL-1β (1ng/ml), cytomix (50ng/ml TNFα, IL-1β and IFNγ) and combined IL-1β+H_2O_2 or cytomix+H_2O_2. Combined treatment with cytomix+H_2O_2 significantly up-regulated the protein expression of IL-27 in the whole cell extract (n=3, paired T test, $p<0.05$). Panel B shows a direct comparison between results from 3 subjects to indicate level of variability.

Figure E1

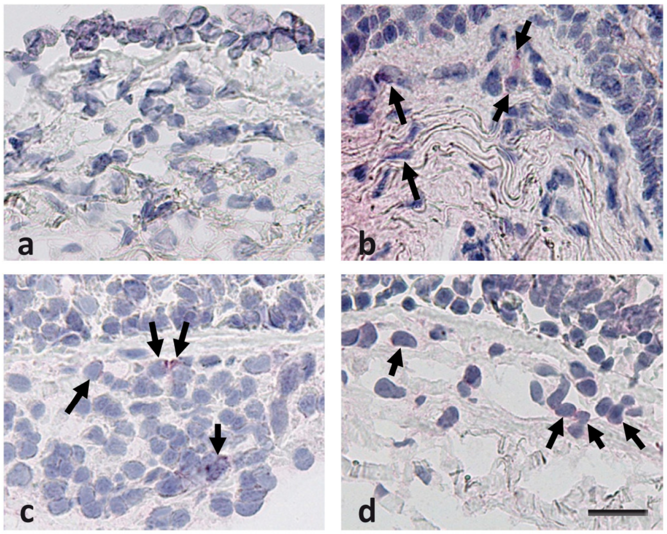

Figure E2

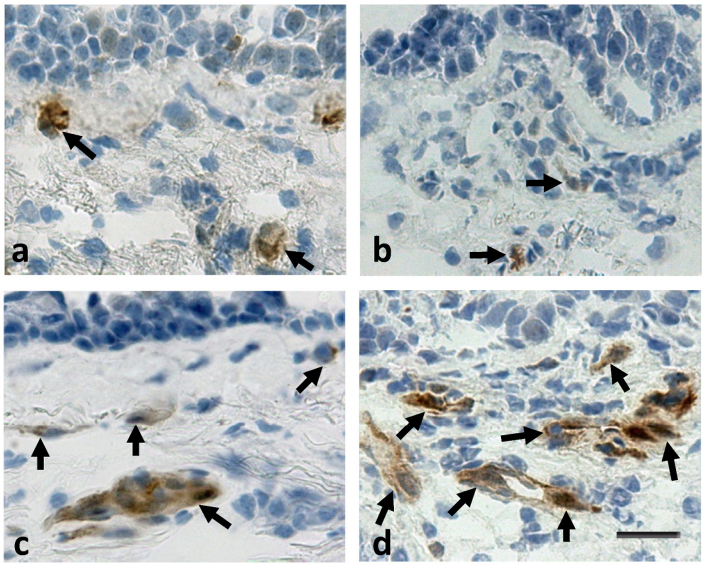

Figure E3

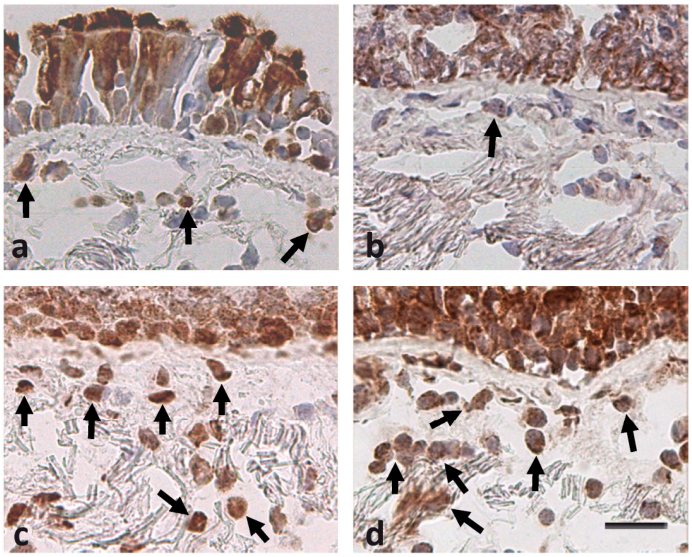

Figure E4

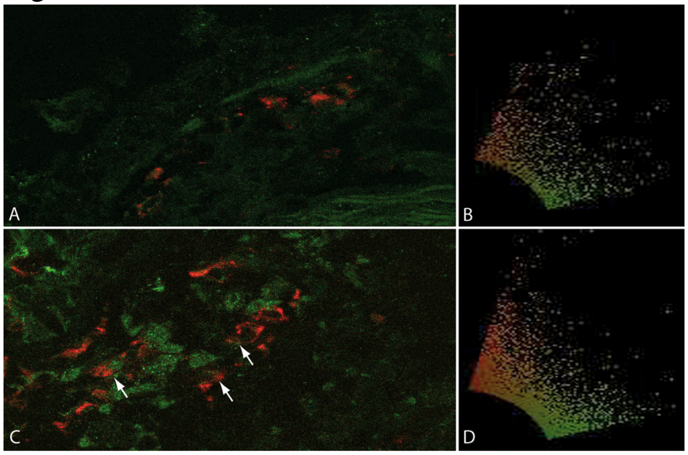

Figure E5

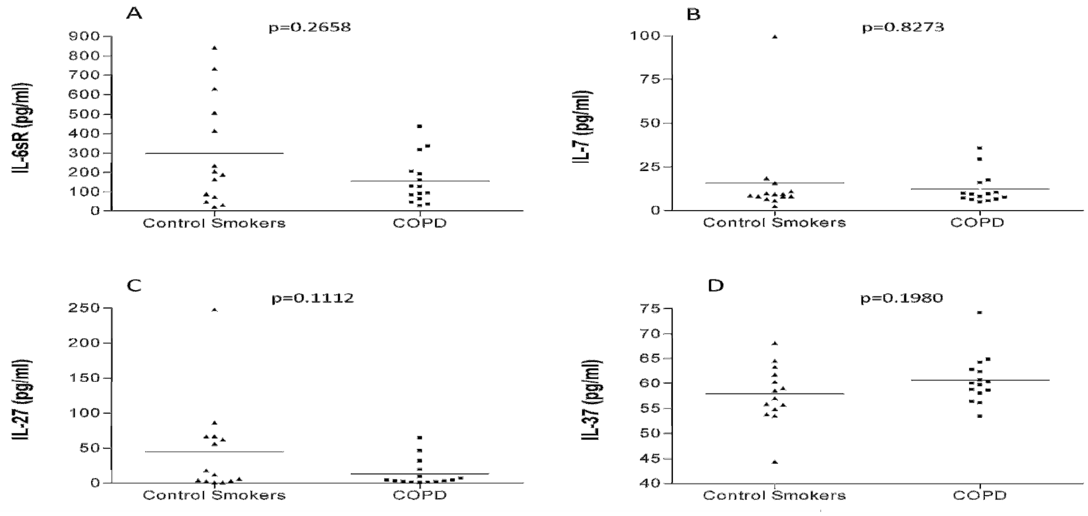

Figure E6

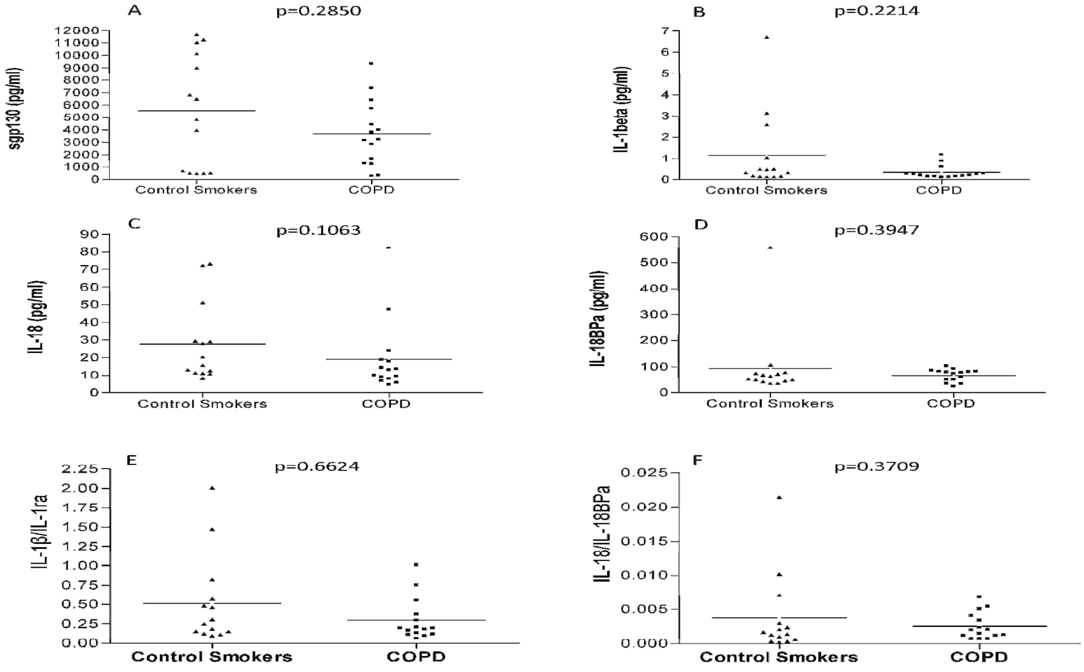

Figure E7

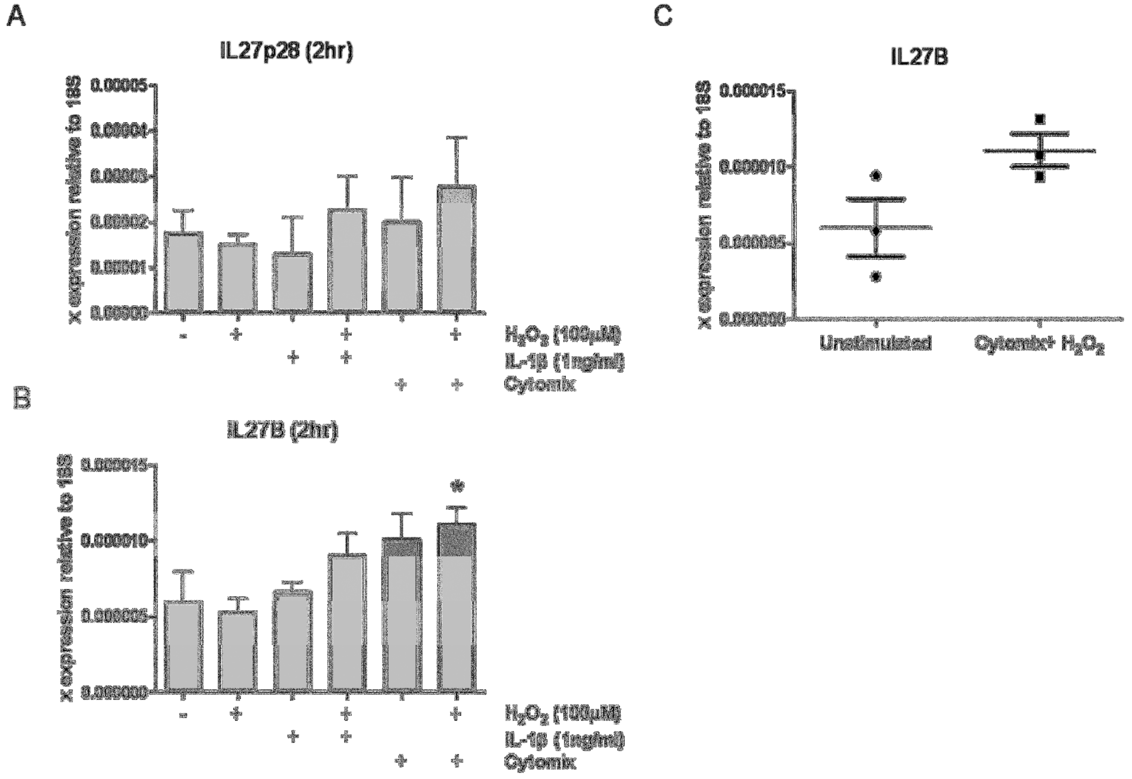

Figure E8

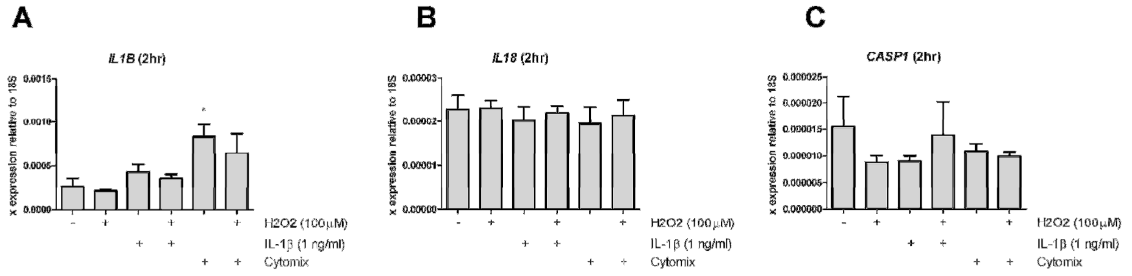

Figure E9

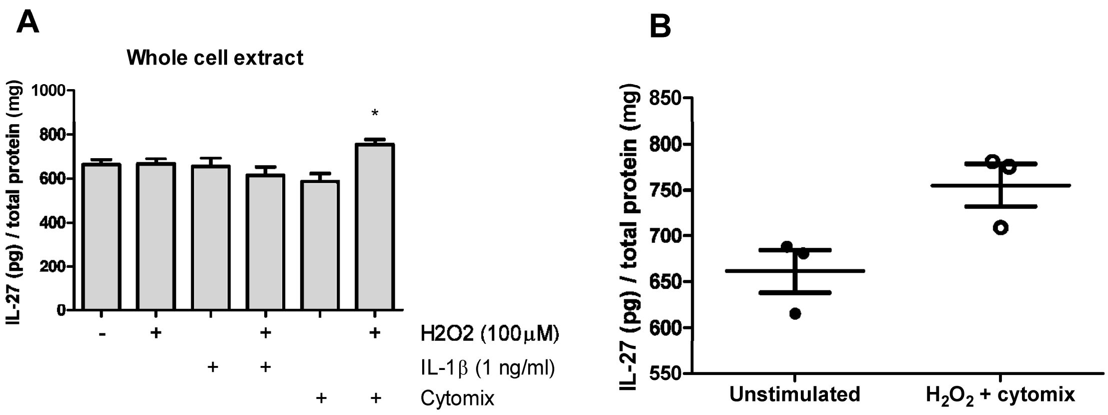
A
Whole cell extract
IL-27 (pg) / total protein (mg)
1000
800
600
400
200
0
*
- + + + H2O2 (100μM)
+ + IL-1β (1 ng/ml)
+ + Cytomix
B
IL-27 (pg) / total protein (mg)
850
800
750
700
650
600
550
Unstimulated
H2O2 + cytomix
